# Dietary patterns and physical inactivity, two contributing factors to the double burden of malnutrition among adults in Burkina Faso, West Africa

**DOI:** 10.1017/jns.2014.11

**Published:** 2014-11-07

**Authors:** Augustin N. Zeba, Hélène F. Delisle, Genevieve Renier

**Affiliations:** 1 Département de Nutrition, Faculté de Médecine, Université de Montréal, C.P. 6128 succ. Centre-ville, Montréal, QC, CanadaH3C 3J7; 2 Institut de Recherche en Sciences de la Santé/Direction Régionale de l'Ouest (IRSS/DRO), 01 BP 545 Bobo Dioulasso 01, Burkina Faso; 3 Centre Hospitalier Universitaire de Montréal, Département de Médecine, Université de Montréal, 1560 Sherbrooke East, Montréal, QC, CanadaH2L 4M1

**Keywords:** Dietary patterns, Physical activity, Micronutrient deficiencies, Cardiometabolic risk factors, Double burden of malnutrition, Adults, Burkina Faso, %BF, percentage body fat, CMRF, cardiometabolic risk factors, HDL-C, HDL-cholesterol, HOMA, homeostasis model assessment, LDL-C, LDL-cholesterol, MET, metabolic equivalent of tasks, MetS, metabolic syndrome, NCD, non-communicable diseases, WC, waist circumference

## Abstract

A population-based cross-sectional study was carried out in the northern neighbourhoods of Ouagadougou (Burkina Faso), to examine the relationship of nutritional deficiencies and cardiometabolic risk factors (CMRF) with lifestyle in adults. We randomly selected 330 households stratified by income tertile. In each income stratum, 110 individuals aged 25–60 years and having lived in Ouagadougou for at least 6 months were randomly selected. We performed anthropometric, dietary intake and physical activity measurements, and blood sample collection. Cluster analysis of dietary intake identified two dietary patterns: ‘urban’ (29 % of subjects) and ‘traditional’ (71 %). The ‘urban’ cluster exhibited a higher intake of fat and sugar, whereas a higher intake of plant protein, complex carbohydrate and fibre was observed in the ‘traditional’ pattern. Female sex, low income and lack of education were associated with the ‘traditional’ cluster, as well as Fe and vitamin A deficiency. CMRF prevalence (abdominal obesity, hypertension, hyperglycaemia, dyslipidaemia) was similar in both clusters. Subjects in the ‘traditional’ cluster spent more time in physical activity and had less sedentary time than those in the ‘urban’ cluster. ‘Traditional’ dietary pattern, low income, female sex and sedentary time were significant contributing factors to the double burden of malnutrition. The rapid nutrition transition is reflected in this co-occurrence of CMRF and nutritional deficiencies. This stresses the need for prevention strategies addressing both ends of the nutrition spectrum.

Risk factors for non-communicable diseases (NCD), including cardiometabolic risk factors (CMRF)^(^^1^^)^ and the metabolic syndrome (MetS), are increasing worldwide and even more rapidly in developing countries^(^^2^^)^. It is widely accepted that CMRF are becoming the leading contributors to the burden of disease, death and disability over the world, and that mortality from NCD is higher and occurs at a younger age in developing than developed countries^(^^2^^,^^3^^)^. Evidence suggests that the nutrition transition^(^^4^^)^, with progressive shifts of diet and lifestyle toward Western patterns, plays a crucial role in the increased prevalence of CMRF^(^^1^^)^ in developing countries. Indeed, there is convincing evidence linking high intake of energy-dense foods and saturated fat, low intake of fruits and vegetables, and sedentary lifestyle with CMRF^(^^5^^)^. The inverse association between physical activity and obesity, blood pressure, insulin sensitivity and lipid profiles is well established^(^^6^^)^. Conversely, a positive relationship between sedentary lifestyle and number of CMRF^(^^7^^)^ has been demonstrated, with WHO estimates suggesting that almost two million deaths per year worldwide are attributable to physical inactivity^(^^8^^)^.

While CMRF are escalating in developing countries, overall and micronutrient malnutrition remains highly prevalent, even among adults in several instances. Available data from sub-Saharan Africa have reported that 18·5, 57·1 and 20 % of women exhibit vitamin A deficiency, anaemia and underweight, respectively^(^^9^^,^^10^^)^. In the adult population of Ouagadougou, we previously reported a high prevalence rate of overweight/obesity (24·2 %), abdominal obesity (12·5 %), hypertension (21·9 %), hyperglycaemia (22·3 %) and low HDL-cholesterol (HDL-C) (30 %), with vitamin A and Fe deficiencies and anaemia being observed in 12·7, 15·4 and 25·5 % of subjects, respectively, predominantly in women compared with men^(^^11^^)^. It has been proposed that low consumption of meat, fish or poultry, especially in poor individuals, is associated with the risk of Fe depletion^(^^12^^–^^14^^)^ which is usually aggregated with other micronutrient deficiencies^(^^15^^)^. It is further argued that poverty could favour diets with adequate energy to meet or exceed energy requirements, while at the same time lacking dietary quality needed to prevent micronutrient deficiencies^(^^16^^,^^17^^)^.

In order to curb the epidemic of CMRF, the UN member states have endorsed, in the High-level Meeting on NCD in 2011, a solid commitment to implement or reinforce programmes to improve populations’ diet and physical activity level, and reduce alcohol consumption^(^^18^^)^. However, this concern for the NCD epidemic should not exclude the issue of nutritional deficiencies, as there is mounting evidence that NCD and undernutrition are linked^(^^19^^,^^20^^)^.

A prerequisite for such programmes is the existence of valid information on population-specific dietary and physical activity patterns, as well as the extent of their association with both CMRF and nutritional deficiencies. Such data are scarce in spite of their relevance in sub-Saharan Africa. The double burden of undernutrition and CMRF has primarily focused on the co-occurrence of maternal obesity and child undernutrition in the same households^(^^21^^,^^22^^)^. As part of a larger project on the nutrition transition and the double burden of under- and overnutrition in West African countries^(^^23^^)^, the present cross-sectional study in Ouagadougou was designed to document CMRF and nutritional deficiencies in adults. We hypothesised that a sedentary lifestyle and a more ‘modern’ diet are associated in an urban setting with both CMRF and micronutrient deficiencies, while controlling for socio-economic factors, featuring the double burden of malnutrition. The present paper reports on the contribution of physical activity and dietary pattern to both CMRF and nutritional deficiencies.

## Methods

### Population and sample

The study was carried out in 2010 in the northern part of Ouagadougou where a population observatory has been in operation since 2008, with periodic collection of socio-economic, sociodemographic and health data in a population sample of 80 000 individuals. This part of the capital city is a vulnerable area on socio-economic and health grounds according to data from national and international institutions^(^^24^^)^. The study sample of 330 subjects aged 25–60 years and stratified by income was selected using the observatory database. The availability of data in this part of Ouagadougou such as household identification, socio-economic and demographic data argued for the present study location. The database included 13 021 households with at least one individual between 25 and 60 years of age. A proxy of household income was derived using principal component analysis, with twelve discriminatory household asset variables (ownership of house, telephone, television, DVD, fridge, motorbike, car, type of household toilet, electricity, type of cooking fuel, and type of floor, roof and walls). Households were split into tertiles of this income proxy. For each tertile, 110 households were randomly selected, with fifty additional subjects as alternates. Only one subject per household was enrolled.

Eligible participants were Burkinabè-born adults aged 25 to 60 years old who had been living in Ouagadougou for at least 6 months and who did not expect to move until the end of the study. Subjects with prior hypertension or diabetes were not excluded from the study. Pregnant or lactating women, as well as physically and mentally disabled subjects, were excluded.

Sample size was determined based on the following information. Previous studies have reported a prevalence of overweight/obesity of 33 %^(^^25^^)^, and a limited access to micronutrient-rich food in 65·6 % of households in Ouagadougou^(^^26^^,^^27^^)^, suggesting an estimated 21·5 % of overweight/obese individuals at risk of micronutrient deficiency. We hypothesised that at least half of them (overall prevalence of 10 %) would show the co-occurrence of overweight/obesity and at least one micronutrient deficiency. A sample size of 300 subjects aged 25 to 60 years was deemed adequate for assessing the prevalence of the double burden of malnutrition with precision of 3 %, statistical power of 80 %, a CI of 95 % and an α error of <0·05, using the software Power Analysis and Sample Size (PASS) supplied by NCSS. The size of the sample was increased by 10 % up to 330, to provide for refusals, missing subjects and incomplete datasets.

### Data collection

The field team consisted of an MD (A. N. Z.), an experienced laboratory technician and two research assistants trained by the first author (A. N. Z.). Following a pre-test of the questionnaire and after obtaining participants’ informed consent, personal interviews provided information on their age, parity, education level, psychosocial factors, and diet and lifestyle patterns. Anthropometric and clinical data as well as blood samples were also collected. The interviews were repeated 6–8 d later for an additional 24 h recall of dietary intake and physical (in)activity.

### Anthropometrics and body composition

Body weight was measured to the nearest 100 g with subjects in light clothing and without shoes, using a portable electronic scale of 150 kg capacity (Seca 803 Clara Scale). Height was measured to the nearest 0·5 cm using a portable locally built stadiometer, with the subject standing upright on a flat surface without shoes, and the back of the heels and the occiput against the stadiometer. Waist circumference (WC) was measured to the nearest 0·1 cm with a flexible non-stretch and tension-regulated steel tape (Gulick measuring tape©; Creative Health Products, Inc.) at the midpoint between the lowest rib and the iliac crest while subjects were standing and breathing normally^(^^28^^)^. The average of two separate measures of body weight, height and WC was used in the analyses. BMI was calculated as weight (kg) divided by height (m^2^). BMI was categorised as follows: underweight: <18·5; normal: 18·5–24·9; overweight, 25–29·9; obese, ≥30 kg/m^2^^(^^29^^)^. Abdominal obesity was defined as a WC ≥ 94 cm for men and ≥80 cm for women^(^^30^^)^. Bioelectrical impedance analysis (BIA) was performed to measure body composition (RJL Systems). For BIA measurements, subjects had to be in the fasting state for at least 12 h, had not engaged in vigorous work or physical activity during the last 24 h and had abstained from alcohol for the previous 48 h. The individual lay on a non-conductive surface with a minimum of clothing before the electrodes were placed on the hand and foot of the same body side (left or right). We computed the percentage body fat (%BF) using the prediction equation for fat-free mass suggested by Sun *et al.* for several race/ethnicity subjects^(^^31^^)^. High body fat was defined as %BF > 25 % in men, and %BF > 33 % in women, as suggested for both black and white subjects^(^^32^^)^.

### Blood pressure

Blood pressure was measured by A. N. Z. with a calibrated anaeroid sphygmomanometer on the right arm of seated subjects after a minimum of 10 min rest. Systolic and diastolic blood pressures were measured twice with an interval of 10 min between the first and the second measurements. Mean of the two readings was used in the analyses. High blood pressure for subjects without prior diagnosis of hypertension was defined as systolic blood pressure ≥130 mmHg or diastolic blood pressure ≥85 mmHg^(^^30^^)^.

### Blood sampling and laboratory measures

Venous blood samples were drawn after an overnight fast of at least 12 h, in 10 ml EDTA and dry tubes for plasma and serum collection, respectively. Blood samples were immediately stored in cold boxes and brought to the laboratory within 2 h. Samples were centrifuged at 3000 rpm for 10 min, sampled in cryotubes and frozen at –32°C. Fasting glucose was immediately determined from plasma samples using the glucose oxidase method at the Medical Analysis Laboratory of the University of Ouagadougou. Hyperglycaemia was defined as fasting plasma glucose >5·6 mmol/l for subjects without prior diagnosis of diabetes^(^^30^^)^. Plasma concentrations of HDL-C, LDL-cholesterol (LDL-C) and TAG were determined by enzymic methods. Cut-offs for low HDL-C were <1·0 mmol/l for men and <1·3 mmol/l for women. The cut-off for high plasma LDL-C was >3·37 mmol/l. Hypertriacylglycerolaemia was defined as plasma TAG concentration >1·7 mmol/l^(^^30^^,^^33^^)^. The ratio of total cholesterol (TC) to HDL-C (TC:HDL-C) was computed and a value >5 for men and >4 for women was defined as high^(^^34^^)^. Serum insulin concentration was measured by RIA (Cisbio Bioassays) and the homeostasis model assessment (HOMA) equation ((fasting glycaemia × serum insulin)/22·5) was used as an index of insulin resistance. Insulin resistance was defined as a HOMA ≥75th centile in the whole study population^(^^35^^)^. Serum retinol was measured using HPLC at the Laboratoire de Chimie Analytique et Toxicologie (LACATOX) de l'Université de Ouagadougou, Burkina Faso, with serum retinol level <0·7 µmol/l being indicative of vitamin A deficiency^(^^36^^)^. Plasma ferritin level was measured using chemiluminescence with a cut-off of <15 µg/l for Fe depletion. Hb was directly measured in the field from a drop of whole blood using HemoCue® (Hemocue HB 201+). Anaemia was defined as Hb <120 g/l in women and <130 g/l in men^(^^37^^)^. Insulin, ferritin and blood lipid determinations were carried out at the Laboratoire de pathologie cellulaire et moléculaire en nutrition, Faculté de Medecine, Nancy-Université, France.

### Nutritional deficiencies and cardiometabolic risk markers

Three nutritional deficiency indicators were considered when assessing the double burden of malnutrition: underweight, Fe depletion and vitamin A deficiency. Individual CMRF were the following, for a maximum count of four: overweight or overall obesity or abdominal obesity; hypertension or under medical treatment; hyperglycaemia or insulin resistance or diagnosed diabetes; and dyslipidaemia (high LDL-C or low HDL-C or hypertriacylglycerolaemia or high TC:HDL-C). The MetS was defined as the clustering within a subject of at least three of the following CMRF: abdominal obesity, hyperglycaemia or treated diabetes, hypertriacylglycerolaemia, low HDL-C, and high blood pressure or treated hypertension^(^^30^^)^.

### Dietary assessment, dietary patterns and micronutrient adequacy ratio

Quantitative dietary intake was assessed with two non-consecutive 24 h recalls carried out by two trained research assistants, using the multiple-pass method for memory bias minimisation^(^^38^^)^. The face-to-face recalls were performed with each participant, separated by 6 or 8 d. To estimate food portion sizes, previously calibrated local kitchen utensils (spoons, glasses, cups, bowls, plates, etc.) were used as visual aids. We computed energy and nutrient intakes using as primary source of data the food composition table developed in the neighbouring country of Mali^(^^39^^)^. Usual nutrient intake was estimated after adjusting for differences between interviewers, and number of days between the recalls, using Software for Intake Distribution Estimation (C-SIDE; Iowa State University)^(^^40^^)^.

A total number of sixteen food groups (local cereals, imported cereals, legumes, oilseeds, tubers, red meat, white meat, fish, eggs, milk and milk products, traditional green leafy vegetables, other vegetables, fruits, local sweetened juices, soft drinks and alcoholic beverages) were defined in order to capture ‘local’ and ‘imported’ food and beverage items. Dietary patterns were generated using K-means cluster analysis ^(^^41^^)^, based on standardised daily intakes (*Z*-scores) of food groups expressed in g per 4184 kJ (1000 kcal)^(^^42^^)^. Two to four cluster solutions were examined to evaluate which set of clusters was meaningful and statistically relevant to define dietary patterns.

The adequacy of intake of eleven micronutrients (vitamin A, thiamin, riboflavin, vitamins B_6_ and B_12_, niacin, folate, vitamin C, Ca, Fe and Zn) was checked against the recommended dietary intake for age and sex^(^^43^^)^. The percentage adequacy was obtained by dividing the intake of a given nutrient by the recommended intake multiplied by 100 and the mean percentage adequacy was computed for each micronutrient. The ‘bioavailability of 5 %’ and ‘low bioavailability’ for Fe and Zn, respectively, were used given the high local phytate load in the diet. We computed micronutrient density (per 4184 kJ (1000 kcal)) for each dietary pattern.

### Physical activity

Physical activity was assessed through two non-consecutives 24 h recalls^(^^44^^)^ by two trained interviewers. Subjects were asked to describe all types of physical activity they performed during the preceding 24 h, classified as time for sleep (during the day and in the night), leisure, transportation and main occupation (employment or housework). Activities for work, transportation and leisure were recorded. These activities were then broken down in two categories according to their intensity according to the compendium of physical activities^(^^45^^)^ as follows: activities <3·0 metabolic equivalent of tasks (MET) and activities ≥3·0 MET. Time spent in each category of activity was recorded in minutes and was expressed as mean number of hours per d. We defined as ‘sedentary time’ time that was spent in activities <3·0 MET, and as ‘active time’ time that was spent in activities ≥3·0 MET and the total daily ‘sedentary’ and ‘active’ hours were computed.

### Statistical analyses

Data were analysed using SPSS version 18·0 (IBM). Comparisons between women and men, and between dietary clusters, were performed using Student's *t* test, or the one-way ANOVA test for means with the Bonferroni *post hoc* test for multiple comparisons, and χ^2^ for proportions. Multiple linear regression models were constructed to analyse the relationship between sociodemographic factors, physical activity and dietary patterns and micronutrient deficiencies and CMRF. A binary logistic regression analysis was performed to assess the odds of having micronutrient deficiencies or CMRF according to ‘urban’ or ‘traditional’ dietary pattern. A multinomial logistic regression with a main-effects model was performed to assess the odds of presenting the double burden of malnutrition phenotypes according to physical activity and dietary patterns while controlling for confounding factors. The level of statistical significance was a *P* value <0·05.

### Ethical considerations

The present study was conducted according to the guidelines laid down in the Declaration of Helsinki and all procedures involving human subjects were approved by the Ethics Committee of the Faculty of Medicine, University of Montreal, and the Ethics Committee for Health Research of Burkina Faso.

The study objectives were clearly explained to participants, selected household heads and local authorities. A written informed consent was obtained from each study subject before enrolment. Participants were informed of their results on blood pressure and glycaemia, and those with abnormal values were referred for diagnosis and treatment, with support by the research project.

## Results

A total of 310 subjects (51·9 % women) completed the study, giving a response rate of 94 %. Mean age of the population under study was 36·4 (sd 9·1) years. Women were less educated than men (*P* = 0·05) and showed lower serum retinol and ferritin, and lower plasma Hb than men (*P* = 0·002, <0·001 and <0·001, respectively) (Table 1). Women had higher BMI values and higher body fat than men (both *P* < 0·001) and their mean HDL-C values were significantly higher than men (*P* = 0·006). Mean WC and other biochemical values were similar in men and women.

**Table 1.** Sociodemographic characteristics and health related markers of the study subjects* (Mean values and standard deviations or percentages and 95 % confidence intervals)

	All (*n* 310)		Women (*n* 161)		Men (*n* 149)		
	Mean	sd	Mean	sd	Mean	sd	*P*†
Age (years)	36·4	9·1	35·7	8·9	37·1	9·2	0·161
Formal education							
None							0·061
%	40·3		45·3		34·9		
95 % CI	34·8–45·8		37·6–53·0		27·3–42·5		
Elementary school							0·913
%	19·0		19·3		18·8		
95 % CI	14·6–23·4		13·2–25·4		12·5–25·1		
High school and above							0·050
%	40·6		35·4		46·3		
95 % CI	35·1–46·1		28·0–42·8		38·3–54·3		
Hb (g/l)	134	19	124	14	145	18	<0·001
Ferritin (μg/l)	66·6	70·9	43·4	50·8	91·7	80·6	<0·001
Retinol (μmol/l)	1·2	0·5	1·1	0·5	1·3	0·5	0·002
BMI (kg/m^2^)	22·7	4·2	23·7	4·8	21·7	3·1	<0·001
WC (cm)	78·4	10·2	79·2	11·5	77·4	8·7	0·127
Body fat (kg)	15·9	13·2	19·9	16·6	11·6	5·9	<0·001
SBP (mmHg)	12·1	1·6	12·1	1·6	12·3	1·6	0·392
DBP (mmHg)	7·5	0·8	7·5	0·8	7·5	0·9	0·763
Glycaemia (mmol/l)	5·4	1·6	5·4	1·7	5·4	1·6	0·754
LDL-C (mmol/l)	2·3	0·8	2·3	0·8	2·3	0·7	0·573
HDL-C (mmol/l)	1·1	0·3	1·1	0·3	1·0	0·2	0·006
TC:HDL-C	3·5	0·9	3·5	0·9	3·6	0·8	0·168
TAG (mmol/l)	0·7	0·3	0·7	0·3	0·7	0·3	0·201
HOMA	5·8	6·1	5·8	4·5	5·9	7·5	0·812

WC, waist circumference; SBP, systolic blood pressure; DBP, diastolic blood pressure; LDL-C, LDL-cholesterol; HDL-C, HDL-cholesterol; TC, total cholesterol; HOMA, homeostasis model assessment.

* Exclusion of eight and two subjects with prior diagnosis of hypertension and diabetes, respectively.

† Significant difference between women and men as determined by Student's *t* test and χ^2^ test.

### Physical activity and dietary patterns

As shown in Table 2, active time (h per d) was significantly lower than sedentary time (*P* < 0·001) in the overall sample. Compared with women, men had less sedentary time, whereas both men and women had similar active times. Active time significantly decreased from low to high income level while sedentary time was significantly higher in the high-income group. More-educated subjects exhibited significantly less active time than uneducated, whereas sedentary time was not different according to education level.

**Table 2.** Physical activity and dietary patterns according to sociodemographic characteristics* (Mean values and standard deviations or percentages and 95 % confidence intervals)

	Daily physical activity by intensity level according to MET (h/d)						Dietary patterns				
	Active time (≥3 MET)			Sedentary time (<3 MET)			‘Urban’ diet (*n* 89)		‘Traditional’ diet (*n* 218)		
	Mean	sd	*P*†	Mean	sd	*P*†	%	95 % CI	%	95 % CI	*P*†
Sample‡	4·4	2·4		11·5	3·1		29·0	23·9, 34·1	71·0	65·9, 76·1	<0·001
Women	4·4	1·9		12·1	2·8		39·3	29·1, 49·5	57·3	50·6, 64·0	0·004
Men	4·3	2·7	0·653	10·8	3·4	<0·001	60·7	50·5, 70·9	42·7	36·0, 49·4	0·004
Income level											
Low	5·3^a^	2·7		10·7^a^	3·3		19·1	11·1, 27·3	41·3	34·8, 47·8	0·002
Middle	4·3^b^	2·2		11·4^a,b^	3·1		37·1	27·1, 47·1	32·1	25·9, 38, 3	0·402
High	3·4^c^	1·6	<0·001	12·4^c^	2·8	<0·001	43·8	33·5, 54·1	26·6	20·7, 32·5	0·003
Formal education											
None	4·8^a^	2·4		11·1	3·2		24·7	15·8, 33·6	46·8	40·2, 53·4	<0·001
Elementary school	4·6^a,b^	2·5		11·6	3·1		15·7	8·2, 23·2	20·6	15·2, 26·0	0·321
High school and above	3·8^b,c^	2·1	0·002	11·7	3·0	0·251	59·6	49·4, 69·8	32·6	26·4, 38·7	<0·001

MET, metabolic equivalent of tasks.

^a,b,c^ Values within a column with unlike superscript letters were significantly different (one-way ANOVA).

* Exclusion of three subjects (one female and two males) in a third cluster of dietary patterns.

† Significant difference between groups as determined by Student's *t* test, χ^2^ test or one-way ANOVA with the Bonferroni *post hoc* test for multiple comparisons.

‡ For the whole sample, sedentary time was significantly higher than active time (*P* < 0·001).

The three-cluster solution of dietary patterns was retained but two patterns remained after excluding three subjects in the third cluster. Table 3 presents food group consumption (per 4184 kJ (1000 kcal)) according to dietary patterns. The ‘urban’ diet cluster (29 % of subjects) was characterised by a significantly higher intake of imported cereals, oilseeds, red meat, eggs, milk and milk products, vegetables other than greens, fruits, local sweetened juices (ginger, tamarind or sweet pea juices) and soft drinks, whereas the ‘traditional’ diet cluster (71 % of subjects) was characterised by a higher intake of local cereals, legumes and traditional green leafy vegetables. As shown in Table 2, female sex, low-income and no-education subjects were significantly aggregated in the ‘traditional’ diet cluster (*P* = 0·004, 0·002 and <0·001, respectively), whereas men, higher-income and more-educated subjects were significantly more represented in the ‘urban’ diet cluster.

**Table 3.** Food group intakes according to dietary pattern (Mean values and standard deviations)

	Intake according to dietary pattern (g/4184 kJ (1000 kcal))						
	Sample (*n* 307)		‘Urban’ diet (*n* 92)		‘Traditional’ diet (*n* 218)		
Food groups	Mean	sd	Mean	sd	Mean	sd	P*
Local cereals	359·2	216·2	234·6	191·7	410·0	205·0	<0·001
Imported cereals	90·9	83·9	155·4	96·8	64·6	61·0	<0·001
Legumes	95·9	112·1	52·7	96·3	113·5	113·6	<0·001
Oil seeds	55·4	61·9	91·1	74·1	40·8	49·4	<0·001
Tubers	10·7	42·6	10·4	47·4	10·9	40·7	0·935
Red meat	11·9	37·9	34·2	64·4	2·9	10·7	<0·001
White meat	0·5	5·6	0·7	7·0	0·3	5·0	0·576
Fish	10·7	26·5	12·2	20·2	10·1	28·7	0·538
Eggs	0·6	4·5	1·9	8·1	0·1	0·6	0·001
Milk and milk products	22·9	71·9	62·3	113·6	6·7	333·9	<0·001
Traditional green leafy vegetables	59·5	74·2	37·6	72·3	68·4	73·3	0·001
Other vegetables	20·1	51·6	50·7	81·9	7·7	22·5	<0·001
Fruits	10·1	33·7	28·4	53·3	2·6	16·1	<0·001
Local sweetened juices	34·6	101·8	68·1	146·9	20·9	72·3	<0·001
Soft drinks	1·9	15·7	6·6	28·8	0·1	1·5	0·001
Alcoholic beverages	25·6	114·8	12·8	60·7	30·8	130·4	0·213

* Significant difference between groups as determined by Student's *t* test.

Micronutrient density (intake per 4184 kJ (1000 kcal)) was significantly higher in the ‘urban’ than ‘traditional’ diet when considering riboflavin, vitamin B_6_, vitamin B_12_, niacin and vitamin C intake. In contrast, folate and Fe density was higher in the ‘traditional’ diet (Table 4). Mean micronutrient adequacy was under 60 % of the recommended intake in both dietary pattern groups for vitamin A, riboflavin, vitamin B_6_, niacin, folate and Ca. Adequacy of vitamin B_12_ was particularly low (only 19 %) in the ‘traditional’ diet cluster, compared with 82 % in the ‘urban’ diet cluster. Similarly, vitamin C adequacy was significantly lower in the ‘traditional’ than in the ‘urban’ cluster (60 *v.* 78 %, respectively; *P* < 0·001). Thiamin, Fe and Zn adequacy was significantly higher in the ‘traditional’ diet cluster as compared with the ‘urban’ one.

**Table 4.** Micronutrient density and adequacy ratio according to dietary pattern (Mean values and standard deviations)

Micronutrients (with FAO/WHO recommended intake)	Micronutrient density of the diet (per 4184 kJ (1000 kcal))						Mean % micronutrient adequacy					
	Total (*n* 307)		Urban diet (*n* 89)		Traditional diet (*n* 218)		P*	Urban diet (*n* 89)		Traditional diet (*n* 218)		P*
	Mean	sd	Mean	sd	Mean	sd		Mean	sd	Mean	sd	
Vitamin A (μg RE)												
500 for females	73·76	46·11	69·46	38·08	75·51	48·99	0·297	26·56	19·13	31·19	19·67	0·060
600 for males												
Thiamin (mg)												
1·1 for females	0·42	0·09	0·44	0·07	0·42	0·09	0·140	78·05	19·21	84·45	22·74	0·020
1·2 for males												
Riboflavin (mg)												
1·1 for females	0·25	0·06	0·27	0·09	0·24	0·05	<0·001	48·14	20·82	46·89	12·69	0·522
1·3 for males												
Vitamin B_6_ (mg)												
1·3 for females 19–50 years												
1·5 for females ≥51 years	0·19	0·06	0·22	0·05	0·18	0·05	<0·001	35·72	11·37	31·59	10·56	0·003
1·3 for males 19–50 years												
1·7 for males ≥ 51 years												
Vitamin B_12_ (μg)												
2·4 for males and females	0·39	0·92	0·89	1·58	0·19	0·19	<0·001	82·32	149·11	18·96	21·11	<0·001
Niacin (mg)												
14 for females	3·36	0·69	3·73	0·74	3·21	0·60	<·0001	51·01	14·66	49·42	11·37	0·310
16 for males												
Folate (μg)												
400 or males and females	79·24	33·18	73·42	23·85	81·61	36·09	0·050	37·89	14·11	46·68	21·66	<0·001
Vitamin C (mg)												
45 for males and females	13·31	5·71	16·85	5·67	11·87	5·07	<0·001	77·73	29·41	59·81	26·44	<0·001
Ca (mg)												
1000 for females 19–50 years												
1300 for females ≥51 years	136·29	41·21	133·08	39·65	137·60	41·84	0·383	27·26	10·02	30·92	10·03	0·004
1000 for males												
Fe (mg)†												
58·8 for females 19–50 years												
22·6 for females ≥51 years	13·29	3·77	10·81	2·57	14·30	3·72	<0·001	67·29	31·32	85·96	41·04	0·001
27·4 for males												
Zn (mg)‡												
9·8 for females	3·88	0·61	3·81	0·63	3·91	0·61	0·208	65·31	18·53	78·85	19·33	<0·001
14·0 for males												

RE, retinol equivalent.

* Significant difference between groups as determined by Student's *t* test.

† 5 % of bioavailability.

‡ Low bioavailability.

The ‘urban’ diet was characterised by a higher amount and percentage of fat and sugar, whereas the ‘traditional’ diet was significantly higher in protein (primarily plant protein), carbohydrate and fibre (Table 5). The percentage contribution of protein to total energy was similar. Total energy intake was significantly higher in the ‘traditional’ diet cluster (*P* < 0·001). The contribution of sugar to the energy intake for both ‘urban’ and ‘traditional’ diets was above the WHO^(^^46^^)^ recommendation for NCD prevention and significantly higher in the urban diet than in the ‘traditional’ one. Fibre intake was lower than WHO recommendations in both diet types^(^^46^^)^.

**Table 5.** Macronutrient intakes and total energy according to dietary pattern (Mean values and standard deviations)

	Intake according to dietary patterns						
	Sample (*n* 307)		Urban diet (*n* 89)		Traditional diet (*n* 218)		
	Mean	sd	Mean	sd	Mean	sd	P*
Protein (g/d)	56·1	1056·9	52·9	1156·3	5756·5	1056·5	056·001
Protein energy (%)	1056·2	156·4	1056·3	156·1	1056·1	156·4	056·377
Fat (g/d)	4356·0	1456·5	4556·7	1556·2	4156·9	1456·2	056·034
Fat energy (%)	1756·4	456·9	1956·6	456·6	1656·4	456·6	<056·001
Carbohydrate (g/d)	41356·8	10156·1	37056·8	8456·9	43156·7	10256·0	<056·001
Carbohydrate energy (%)	7356·8	7·1	7156·6	756·0	7456·7	656·9	<056·001
Sugar (g/d)	10656·4	11456·7	13056·4	9856·3	9656·6	11956·6	<056·019
Sugar energy (%)	1756·6	1556·8	2456·1	1756·6	1456·9	1456·3	<056·001
Fibre (g/d)	1756·9	556·8	1456·5	356·9	1956·3	556·9	<056·001
Total energy							<056·001
kJ/d	942056·7	229456·9	869956·0	197956·9	971456·8	235356·5	
kcal/d	225156·6	54856·5	207956·1	47356·2	232156·9	56256·5	

* Significant difference between groups as determined by Student's *t* test.

### Malnutrition and cardiometabolic risk factors according to physical activity and dietary patterns

Anaemic, Fe-deficient and vitamin A-deficient subjects were more prone to be aggregated in the traditional diet cluster: OR 1·85 (95 % CI 1·01, 3·43; *P* = 0·049), OR 3·27 (95 % CI 1·33, 8·03; *P* = 0·009) and OR 3·21 (95 % CI 1·21, 8·49; *P* = 0·019), respectively. The OR of CMRF were not associated with dietary patterns (Table 6), but subjects exhibiting overweight/obesity, abdominal obesity, high %BF, high glycaemia, high blood pressure, hypertriacylglycerolaemia, MetS, high LDL-C or insulin resistance (high HOMA) had significantly fewer active hours. Sedentary time was also significantly higher in subjects with overweight/obesity, abdominal obesity, high %BF and the MetS.

**Table 6.** Micronutrient deficiency and cardiometabolic risk factors according to physical activity and dietary pattern (Means values and standard deviations, numbers of subjects and odds ratios)

	Daily physical activity by intensity level according to MET (h/d)						Dietary patterns: odds of risk in ‘traditional’ *v.* ‘urban’ pattern				
	Active hours (>3 MET)			Sedentary hours (≤3 MET)			‘Urban’ diet	‘Traditional’ diet			
	Mean	sd	P*	Mean	sd	P*	(*n* 89)	(*n* 218)	OR	95 % CI	P*
Micronutrient deficiency markers											
Hb status											
Non-anaemic (*n* 228)	4·2	2·4		11·3	3·3		73	155	1·00		
Anaemic (*n* 79)	4·7	2·2	0·157	11·8	2·7	0·209	16	63	1·85	1·0, 3·4	0·049
Fe status											
Normal Fe (*n* 260)	4·3	2·5		11·3	3·2		83	177	1·00		
Deficient (*n* 47)	4·5	1·8	0·736	12·2	2·5	0·082	6	41	3·27	1·3, 8·0	0·009
Vitamin A status											
Normal (*n* 267)	4·3	2·4		11·5	3·1		84	183	1·00		
Deficient (*n* 40)	4·9	1·9	0·100	10·9	3·0	0·287	5	35	3·21	1·2, 8·5	0·019
Cardiometabolic markers											
BMI											
18·5–24·9 (*n* 203)	4·6^a^	2·5		11·1^a^	3·1		55	148	1·00		
< 18·5 (*n* 30)	4·5^a,b^	2·8		11·1^a,b^	3·4		8	22	1·02	0·4, 2·4	0·961
≥ 25 (*n* 74)	3·6^c^	1·6	0·008	12·5^c^	2·8	0·003	26	48	0·68	0·4, 1·2	0·194
Waist circumference											
Normal (*n* 235)	4·6	2·5		11·2	3·1		65	170	1·00		
Abdominal obesity (*n* 72)	3·7	1·7	0·010	12·4	3·0	0·004	24	48	0·76	0·4, 1·3	0·354
Percentage body fat											
Normal (*n* 235)	4·6	2·5		11·1	3·2		67	167	1·00		
High (*n* 72)	3·6	1·6	0·001	12·6	2·7	<0·001	22	51	0·91	0·5, 1·6	0·753
Glycaemia											
Normal (*n* 201)	4·1	2·2		11·5	2·9		58	143	1·00		
High (*n* 106)	4·7	2·6	0·043	11·3	3·4	0·553	31	75	0·94	0·6, 1·6	0·943
Blood pressure											
Normal blood pressure (*n* 196)	4·7	2·6		11·4	3·2		63	133	1·00		
High blood pressure (*n* 111)	3·8	1·9	0·002	11·6	3·0	0·590	26	85	1·54	0·9, 2·4	0·107
HDL-cholesterol											
Normal (*n* 215)	4·4	2·5		11·2	3·2		67	148	1·00		
Low (*n* 92)	4·3	2·1	0·758	11·9	2·9	0·054	22	70	1·44	0·8, 2·5	0·201
Triacylglycerolaemia											
Normal (*n* 301)	4·4	2·3		11·4	3·1		88	213	1·00		
Hypertriacylglycerolaemia (*n* 6)	2·3	0·3	0·029	12·7	3·0	0·323	1	5	2·06	0·2, 17·9	0·511
Clustering of MetS factors											
Zero factors (*n* 75)	4·9^a^	2·6		10·7^a^	2·9		27	48	1·00		
One factor or two factors (*n* 200)	4·3^a,b^	2·3		11·6^a,b^	3·2		54	146	1·52	0·8, 2·7	0·146
MetS (*n* 32)	3·7^b,c^	1·8	0·043	12·1^b,c^	2·7	0·039	8	24	1·68	0·7, 4·3	0·269
LDL-cholesterol											
Normal (*n* 276)	4·5	2·4		11·4	3·2		81	276	1·00		
High (*n* 31)	3·5	1·8	0·028	12·2	2·8	0·180	8	23	1·19	0·5, 2·7	0·680
Insulin resistance											
No (*n* 229)	4·5	2·5		11·2	3·3		64	165	1·00		
Yes (*n* 78)	3·8	1·8	0·019	12·0	2·7	0·063	25	53	0·82	0·5, 1·4	0·491

MET, metabolic equivalent of tasks; MetS, metabolic syndrome.

* Significant difference between groups as determined by Student's *t* test, χ^2^ test or one-way ANOVA with the Bonferroni *post hoc* test for multiple comparisons.

^a,b,c^ Values within a column with unlike superscript letters were significantly different (one-way ANOVA).

The relationship between micronutrient status or CMRF on the one hand, and physical activity and dietary patterns (‘urban’ = 0 and ‘traditional’ = 1) on the other hand was tested using two regression models. In the first model (without control variables), the ‘urban’ dietary pattern was significantly associated with higher Hb and higher serum ferritin concentrations (β = –0·173 and –0·133; *P* < 0·05, respectively) and showed a borderline association with higher serum retinol (β = –0·103; *P* = 0·076) (data not shown). Regarding CMRF, the ‘urban’ pattern was only significantly associated with high HOMA (β = –0·118; *P* = 0·047). Active time was significantly and negatively associated with BMI, WC, body fat, systolic and diastolic blood pressure, triacylglycerolaemia and LDL-C, whereas sedentary time was significantly and positively associated with BMI, WC and systolic blood pressure. After controlling for sociodemographic characteristics (Table 7), dietary patterns lost their significant predictive power of Hb and serum retinol concentration. This model now reveals a positive and significant association of male sex with Hb, ferritin and retinol concentrations, with female sex being associated with lower values. Income level was also positively associated with Hb and serum retinol concentrations, while sedentary time was negatively associated with serum ferritin level. Controlling for sociodemographic characteristics did not change the significant association of active time with CMRF or between the urban dietary pattern and higher insulin resistance as evidenced by higher HOMA (β = –0·105; *P* = 0·042). Age was positively and significantly associated with BMI, WC, systolic and diastolic blood pressure, triacylglycerolaemia and LDL-C level. Female sex was significantly associated with high BMI, WC, high body fat and high HDL-C. Education level was positively associated with BMI while income level was negatively associated with glycaemia.

**Table 7.** Multiple linear regression models of micronutrient deficiencies and cardiometabolic risk markers on sociodemographic factors, physical activity and dietary patterns

									Micronutrient deficiency marker as dependent variable											
									Hb (g/l)		Ferritin (μg/l)		Retinol (μmol/l)							
							Independent variables		β	*P*	β	*P*	β	*P*						
							Active time (h)		−0·059	0·337	−0·060	0·410	−0·013	0·672						
							Sedentary time (h)		–0·025	0·672	−0·111	0·023	−0·009	0·909						
							Dietary pattern*		−0·059	0·244	−0·071	0·213	−0·038	0·519						
							Age		0·051	0·305	0·105	0·089	0·116	0·043						
							Sex†		0·530	<0·001	0·327	<0·001	0·153	0·008						
							Income level‡		0·106	0·042	0·032	0·812	0·145	0·026						
							Education§		0·033	0·567	0·05	0·065	0·126	0·062						
Independent	Cardiometabolic risk markers as dependent variables																			
variables	BMI		WC		Body fat		Glycaemia		SBP		DBP		Triacylglycer olaemia		HDL-C		LDL-C		HOMA	
	β	*P*	β	*P*	β	*P*	β	*P*	β	*P*	β	*P*	β	*P*	β	*P*	β	*P*	β	*P*
Active time (h)	−0·128	0·034	−0·195	0·005	−0·124	0·045	−0·019	0·795	−0·264	<0·001	−0·210	0·003	−0·233	0·001	0·011	0·880	−0·145	0·042	−0·045	0·551
Sedentary time (h)	0·172	0·006	0·133	0·027	0·031	0·584	0·002	0·978	0·107	0·055	0·093	0·180	0·004	0·959	−0·056	0·423	0·049	0·475	0·032	0·667
Dietary pattern*	−0·071	0·214	−0·039	0·500	−0·019	0·699	−0·052	0·393	0·.078	0·157	0·062	0·288	−0·007	0·911	−0·035	0·559	−0·054	0·362	−0·105	0·042
Age	0·140	0·013	0·246	<0·001	0·071	0·132	−0·017	0·780	0·350	<0·001	0·220	<0·001	0·130	0·025	−0·073	0·212	0·194	0·001	−0·093	0·126
Sex†	−0·280	<0·001	−·0105	0·026	−0·316	<0·001	0·019	0·748	−0·015	0·786	−0·030	0·608	0·047	0·421	−0·181	0·002	−0·069	0·237	−0·011	0·862
Income level‡	0·079	0·211	0·122	0·068	0·025	0·700	−0·117	0·038	0·007	0·909	−0·071	0·276	−0·065	0·321	0·103	0·116	0·110	0·089	0·034	0·625
Education§	0·130	0·041	0·086	0·179	0·065	0·312	−0·079	0·241	0·055	0·367	0·069	0·286	0·088	0·178	0·114	0·084	0·060	0·358	−0·003	0·967

WC, waist circumference; SBP, systolic blood pressure; DBP, diastolic blood pressure; HDL-C, HDL-cholesterol; LDL-C, LDL-cholesterol; HOMA, homeostasis model assessment.

* Dietary pattern (urban dietary pattern = 0; traditional dietary pattern = 1).

† Sex (0 = female; 1 = male).

‡ Income level (low = 0; middle = 1; high = 2).

§ Education (none = 0; elementary school = 1; high school and above = 2).

Taking account of CMRF and nutritional deficiency markers, five phenotypes were identified: phenotype 1 for ‘normal’ subjects showing neither nutritional deficiencies nor CMRF (11·72 %); phenotype 2 or ‘deficient’ subjects, exhibiting underweight or micronutrient deficiencies (6·84 %); phenotype 3 for subjects with CMRF, overweight/obesity or other (55·40 %); phenotype 4 for the first ‘double burden’ group, with subjects exhibiting overweight/obesity plus at least one micronutrient deficiency (8·46 %); and phenotype 5, the second ‘double burden’ group with subjects having CMRF other than overweight/obesity associated with at least one micronutrient deficiency or underweight (17·58 %). Using phenotype 1 (‘normal’ subjects) as the reference group, the multinomial logistic regression showed (Table 8) that subjects with more sedentary time (OR 1·17; 95 % CI 1·02, 1·49; *P* = 0·038) were more likely to be found in phenotype 4 (co-occurrence of overweight/obesity plus micronutrient deficiencies), while men and middle-income subjects were less likely to have this phenotype. Subjects with a ‘traditional’ diet pattern (OR 3·04; 95 % CI 1·01, 9·22; *P* = 0·050) were more likely to present the ‘phenotype 5’ (co-occurrence of other CMRF associated with micronutrient deficiencies or underweight), while men and middle-income subjects were less likely to have this phenotype. Of note, middle-income subjects were at lower odds of all nutritional burden phenotypes, whereas education level showed no association with these phenotypes.

**Table 8.** Multinomial logistic regression of nutrition deficiency and cardiometabolic risk phenotypes on physical activity and dietary patterns controlling for sociodemographic factors (Odds ratios and 95 % confidence intervals)

		Dependent variable: clusters of CMRF with malnutrition marker*											
	Phenotype 1 (‘normal’ subjects)	Phenotype 2 (nutritional deficiencies only)			Phenotype 3 (CMRF only)			Phenotype 4 (double burden)			Phenotype 5 (double burden)		
Independent variables	OR	OR	95 % CI	*P*	OR	95 % CI	*P*	OR	95 % CI	*P*	OR	(95 % CI	*P*
Active time (h)	1	0·97	0·72, 1·29	0·833	0·95	0·78, 1·16	0·670	0·98	0·72, 1·33	0·913	0·92	0·72, 1·16	0·481
Sedentary time (h)	1	0·95	0·76, 1·24	0·851	1·09	1·02, 1·28	0·029	1·17	1·02, 1·49	0·038	1·28	0·99, 1·65	0·059
Dietary pattern													
Urban	1	1			1			1			1		
Traditional	1	1·17	0·32, 4·23	0·978	1·11	0·49, 2·57	0·776	3·48	0·80, 15·12	0·096	3·04	1·01, 9·22	0·050
Age		0·98	0·91, 1·06	0·584	1·04	1·01, 1·10	0·039	1·02	0·95, 1·10	0·514	0·99	0·94, 1·04	0·805
Sex													
Women	1	1			1			1			1		
Men	1	0·49	0·15, 1·59	0·241	0·49	0·20, 0·97	0·049	0·16	0·04, 0·58	0·005	0·36	0·13, 0·95	0·041
Income level													
Low	1	1			1			1			1		
Middle	1	0·13	0·03, 0·65	0·012	0·47	0·18, 0·94	0·046	0·34	0·07, 0·93	0·045	0·32	0·11, 0·92	0·034
High	1	0·76	0·15, 4·00	0·752	0·95	0·28, 3·24	0·939	0·58	0·07, 2·06	0·506	0·31	0·07, 1·33	0·116
Education													
None	1	1			1			1			1		
Elementary school	1	0·737	0·706, 2·24	0·7283	1·726	0·743, 3·42	0·7650	0·761	0·714, 2·64	0·7508	0·787	0·728, 2·71	0·7806
High school and above	1	1·24	0·28, 5·39	0·774	2·46	0·92, 6·59	0·073	1·42	0·35, 5·77	0·622	1·11	0·34, 3·59	0·863

CMRF, cardiometabolic risk factors; phenotype 1, ‘normal’ subjects with neither cardiometabolic risk factor nor nutritional deficiencies; phenotype 2, subjects with micronutrient deficiencies or underweight; phenotype 3, subject with ‘CMRF’ only, i.e. overweight/obesity associated or not to other CMRF; phenotype 4, ‘double burden’ subjects with a co-occurrence of overweight/obesity and micronutrient deficiencies within the same individual; phenotype 5, ‘double burden’ subjects with a co-occurrence of other CMRF with micronutrient deficiencies or underweight.

* Phenotype 1, ‘normal’ subjects (with neither cardiometabolic risk factor nor nutritional deficiencies) as the reference group for the dependent variable.

## Discussion

In this cross-sectional study in an adult population of Ouagadougou, we identified two dietary patterns using cluster analysis. This approach, by addressing nutrient interactions and considering diet as a whole rather than focusing on specific foods or nutrients, is more and more valued in epidemiological studies^(^^47^^)^. The ‘urban’ dietary pattern was indeed more city-like because of higher consumption of soft drinks, non-traditional vegetables and imported cereals such as rice and wheat products, which are typically eaten in urban areas. The ‘traditional’ dietary pattern was predominantly characterised by higher intake of traditional green leafy vegetables and local cereals (millet and maize) typical of rural areas.

We did not observe in the present study population an advanced stage of dietary transition with Westernised^(^^4^^,^^48^^)^ food habits characterised by high intake of processed foods, animal products and other foods high in fat, and sweets as substitutes for traditional foods. However, we documented in 29 % of the subjects an ‘urban’ dietary pattern with a more diversified diet according to the number of food groups consumed, and with imported foods being added to the ‘traditional’ diet base. Similar findings were reported in Benin^(^^49^^)^, a neighbouring country, where the ‘transitional’ diet consisted of a ‘traditional’ diet plus added Western foods which do not displace traditional foods, at least not as yet. While the ‘urban’ diet in Ouagadougou was higher in fat and sugar that the traditional diet, fat intake was low in both dietary patterns as it did not reach 20 % of total energy intake. In contrast, sugar intake was high, representing well above 10 % of total energy as recommended by WHO^(^^29^^)^. While the ‘traditional’ diet was higher in fibre than the ‘urban’ diet, it was still lower than the recommended 25 g/d.

Higher-income, more-educated and male subjects were significantly aggregated in the ‘urban’ diet cluster, while there were proportionally more lower-income, non-educated and female subjects in the ‘traditional’ diet cluster. These data are in agreement with several previous studies showing an association between dietary patterns and socio-economic characteristics^(^^49^^–^^51^^)^. Studies on dietary patterns in different populations such as Brazil^(^^52^^)^, Korea^(^^51^^)^ and Greece^(^^53^^)^ usually documented the presence of ‘traditional’ dietary patterns (based on staples and other traditional foods), as well as modified dietary patterns characterised by highly processed foods, refined sugar and grains, and abundance of energy-dense and micronutrient-poor foods. In the present study, both dietary patterns had a low micronutrient density, with a micronutrient adequacy below 60 % of the recommended intake for six out of eleven micronutrients. The significant aggregation of anaemic, Fe-deficient and vitamin A-deficient subjects in the ‘traditional’ diet cluster, in spite of the higher percentage adequacy for Fe and vitamin A in this diet, may reflect the lower bioavailability of Fe and lower vitamin A activity of plant sources of carotenoids in the cereal- and green leafy-based ‘traditional’ diet, compared with the ‘urban’ diet that includes more meat.

The present results further demonstrate that the ‘urban’ dietary pattern was significantly and independently associated with insulin resistance. While dietary fat intake, particularly saturated fat, has been shown to be associated with insulin resistance^(^^54^^–^^56^^)^, a recent and extensive review on the relationship between carbohydrate intake and insulin resistance^(^^57^^)^ could not demonstrate a clear and consistent association. This is in line with our findings, as the ‘urban’ diet exhibited significantly higher intake of fat and fat contribution to energy, whereas the ‘traditional’ diet had higher intake of carbohydrate. The shift toward ‘urban’ or even ‘Western’ dietary patterns will probably continue and the present findings on the association of the ‘urban’ diet with insulin resistance is of particular concern, as it is in line with the International Diabetes Federation^(^^58^^)^ projections on diabetes increase in developing countries until 2030. The unexpected association between high glycaemia and low income is another matter for concern, as this metabolic risk factor seems to no longer be a problem of affluent individuals only. A closer look shows that 63·2 % (*n* 68) of high glycaemia subjects (55 % of them belong to the low-income group) were not insulin resistant, i.e. these subjects exhibited lower levels of insulin while having high glycaemia; otherwise they would have been in the insulin-resistant group. Among these non-insulin-resistant and high glycaemia subjects, 15 % were underweight and only 20·6 % were overweight/obese. Such results legitimate the questioning raised in previous reports^(^^59^^,^^60^^)^ about the insulin-requiring diabetes or malnutrition-related diabetes in African populations and stress the need for more African studies to investigate this specific issue further.

In contrast^(^^61^^–^^63^^)^ or in agreement^(^^51^^,^^64^^–^^66^^)^ with other studies, we observed no statistical difference in the OR of overweight/obesity or abdominal obesity according to dietary clusters. Energy intake was significantly higher in the ‘traditional’ cluster, but so was physical activity (‘active’ time in the present study), very much like several other studies showing that rural individuals with traditional diets have higher energy intake and also higher energy expenditure than their urban counterparts.

As expected, subjects with overweight/obesity, abdominal obesity, higher body fat, high glycaemia, high blood pressure, high triacylglycerolaemia, high LDL-C, insulin resistance and those with the MetS had significantly less active time, and also consistently showed higher sedentary time (except for high glycaemia, high blood pressure, high triacylglycerolaemia, LDL-C and insulin resistance). Daily physical activity has consistently been shown to reduce abdominal or overall obesity, and improve glucose homeostasis, insulin sensitivity, blood pressure and circulating lipoprotein profile, whether in developing^(^^6^^,^^67^^–^^69^^)^ or developed^(^^70^^,^^71^^)^ country populations. The central role of physical activity in CVD prevention^(^^72^^,^^73^^)^ is widely recognised. Several African studies^(^^68^^,^^69^^,^^74^^)^ have reported that rural populations have higher physical activity than their urban counterparts, with high-income groups in urban areas being less active than low-income ones. Consistent with these findings, the present results portrayed significantly higher sedentary time in women, high-income, as well as in more-educated subjects. Deterrents to physical activity such as the low status and lack of promotion of physical activity, as well as the absence of sidewalks and insecurity of walking, may be at play in urban Africa and need to be addressed in order to halt the progression of NCD.

We also reported an independent and significant association between sedentary time and Fe deficiency. Keeping in mind that sedentary time is significantly higher in overweight/obese subjects, this finding supports a possible increased risk of Fe deficiency in overweight/obese individuals^(^^75^^–^^77^^)^, a condition that could therefore promote the double burden of malnutrition.

We have used in the present paper more sensitive cut-offs for high blood pressure, glycaemia and WC^(^^30^^)^ than in a previous paper^(^^11^^)^ and we report a 26 % double burden of malnutrition, compared with 23·5 %^(^^11^^)^. Generic WC cut-offs used may not be appropriate for Africans, as recently reported^(^^78^^)^. Sedentary time, low income status and the ‘traditional’ dietary pattern (borderline) appeared to be associated with the co-occurrence of overweight/obesity plus micronutrient deficiencies (phenotype 4). Sedentary time (borderline), low income status, female sex and ‘traditional’ dietary pattern were also significantly associated with the co-occurrence of other CMRF plus underweight or micronutrient deficiencies (phenotype 5). Given the low micronutrient density of the ‘traditional’ diet, it is not surprising that this dietary pattern was associated with the two phenotypes of double burden, because these imply micronutrient deficiencies in addition to CMRF. Middle-income subjects appeared less affected by micronutrient deficiencies, CMRF and their co-occurrence. One should note that middle-income subjects were aggregated without statistical difference in both ‘urban’ and ‘traditional’ diet clusters, with active time significantly lower than that of the low-income group but also higher than that of the high-income group. These findings suggest that the middle-income subjects may be less exposed to two kinds of risk: the deficiencies experienced by the poor and the unhealthy lifestyles of more affluent. The variable most consistently associated with overweight/obesity in the present study was sedentary time, explaining the association of a sedentary lifestyle with phenotype 4 of double burden and a borderline association with phenotype 5 of double burden. One should keep in mind that poverty and female sex were strongly associated with the ‘traditional’ diet pattern. This dietary pattern was less diversified, a common feature of food insecurity, which is known to be more prevalent among the poor and particularly among women^(^^16^^,^^79^^,^^80^^)^. In fact, almost 52 % of Ouagadougou^(^^81^^)^ households reportedly experience chronic food insecurity. These findings fit with the hypothesis that in food-insecure households, cheap and energy-dense foods may contribute to overweight in adults, while not sustaining a healthy nutritional status due in part to inadequate micronutrient density^(^^17^^,^^82^^,^^83^^)^. In such context, having enough food to ward off hunger is the main concern of individuals.

Several limitations can be identified in the present study. The cross-sectional design does not allow any inference to be drawn with regard to causal relationships. Furthermore, the study is only representative of one district in Ouagadougou and the results cannot be extrapolated to the whole urban population of Burkina Faso without extreme caution. The cluster analysis used in the present study to identify the different food patterns is open to several criticisms: It involves subjective decisions as regards food grouping and cluster labelling. Variations across studies in cluster numbers and characteristics preclude comparisons. Nonetheless, dietary patterns were based on rigorous dietary assessment. We conducted two 24 h dietary recalls using a validated multiple-pass method to reduce memory bias. Additionally, C-SIDE software was used for the reduction of the intra-individual intake variation. Unfortunately, we could not compute the SFA and PUFA and cholesterol intakes that would have been helpful for the assessment of diet healthfulness. Other limitations are that measuring physical activity with 24 h recalls is a method that has not been validated as yet. Finally, the duration of the data collection period (from January to July) is another limitation, as seasonal variations cannot be captured while they may confound some findings.

Despite these limitations, the present study provides useful data on the ongoing nutrition transition in Ouagadougou and reports for the first time the coexistence of malnutrition and nutrition-related NCD risk factors in the same individual urban adults. Indeed, our data clearly show that a nutrition transition is underway in this adult population of Ouagadougou, affecting both food and physical activity patterns. Interestingly, the nutrition transition, rather than promoting an isolated rise in CMRF, fosters the double burden of malnutrition because deficiency conditions persist. Concerted efforts at national and international levels are compelling in low-income countries in order to halt the NCD epidemic while also addressing nutritional deficiencies.
